# Genetic dissection on rice grain shape by the two-dimensional image analysis in one *japonica* × *indica* population consisting of recombinant inbred lines

**DOI:** 10.1007/s00122-015-2560-7

**Published:** 2015-07-02

**Authors:** Changbin Yin, Huihui Li, Shanshan Li, Lidong Xu, Zhigang Zhao, Jiankang Wang

**Affiliations:** The National Key Facility for Crop Gene Resources and Genetic Improvement, Institute of Crop Science and CIMMYT China Office, Chinese Academy of Agricultural Sciences, Beijing, 100081 China; National Key Laboratory for Crop Genetics and Germplasm Enhancement, Research Center of Jiangsu Plant Gene Engineering, Nanjing Agricultural University, Nanjing, 210095 China

## Abstract

***Key message*:**

**This article used seven characters from the 2D image analysis to dissect the genetic architecture underlying rice grain shape in one*japonica*** × ***indica*population consisting of 215 recombinant inbred lines.**

**Abstract:**

Two-dimensional (2D) digital image analysis is efficient for investigating the rice grain shape characters in large genetic and breeding populations. In this study, we used 2D image analysis to investigate seven characters, i.e., grain length (GL), grain width (GW), length-to-width ratio (LW), grain area (GA), grain circumference (GC), grain diameter (GD), and grain roundness (GR), in one *japonica* × *indica* genetic population consisting of 215 recombinant inbred lines. GL and GW can be recorded manually as well, and have been extensively used together with LW (i.e., GL/GW) in genetic studies on grain shape. GC and GA can be hardly measured manually, and have not been used together with GD and GR. Results indicated that the seven characters could be precisely measured by 2D image analysis, genotype by environment interaction was low, and heritability was high. Each character was controlled by a few major stable genes and multiple minor additive genes. A total of 51 QTL were detected for the seven characters across four diverse environments, 22 from GL, GW, and LW, the three traditional characters, and 29 from the other four characters. The 51 QTL were clustered in eighteen marker intervals. Comparing with previous studies and analyzing the stability of identified QTL, we found six non-reported marker intervals, one each on chromosomes 2 and 3, and two each on chromosomes 6 and 8. The newly identified loci and the large-scale phenotyping system would greatly improve our knowledge about the genetic architecture and the future rice breeding on grain shape.

**Electronic supplementary material:**

The online version of this article (doi:10.1007/s00122-015-2560-7) contains supplementary material, which is available to authorized users.

## Introduction

Rice (*Oryza sativa* L.) is a seed-eating cereal crop, and therefore grain shape is a vital appearance quality trait. In addition, rice grain shape is also a key determinant on grain yield (Huang et al. 2013). In genetics, grain shape has been widely accepted as a complex trait controlled by multiple genes with small effects. By phenotyping, it is complex because it could be evaluated in different ways. In conventional rice genetic study and breeding, grain shape is characterized by grain length (GL), grain width (GW), and the length-to-width ratio (LW), which greatly affect grain yield, grain appearance quality, and marketability (Wan et al. 2005; Fan et al. 2006). As a result, lots of QTL have been detected in the past two decades for the three characters relevant to grain shape (Xing et al. 2000; Tan et al. 2000; Li et al. 2003, 2004; Aluko et al. 2004; Wan et al. 2005; Bai et al. 2010; Shao et al. 2010; Huang et al. 2013).

Based on QTL identified with relatively large phenotypic effects, some genes related to grain shape have been isolated and cloned in rice, such as *GW2* (Song et al. 2007), *gw5/qSW5* (Shomura et al. 2008; Weng et al. 2008; Wan et al. 2008), *GIF1/OsCIN2* (Wang et al. 2008, 2010), *srs*-*3* (Tanabe et al. 2007; Kitagawa et al. 2010), *GS5* (Yu et al. 1997; Li et al. 2000, 2011), *GS3* (Fan et al. 2006; Takano-Kai et al. 2009, 2011; Mao et al. 2010; Wang et al. 2011), *qGW8/OsSPL16* (Wang et al. 2012a, b), *SG1* (Nakagawa et al. 2012), and *DEP1/qPE9*-*1* (Yan et al. 2007; Zhou et al. 2009; Huang et al. 2009; Yi et al. 2011; Taguchi-Shiobara et al. 2011; Sun et al. 2014). Positive regulator *PGL1* and *PGL2* were found in the network of grain shape genes (Heang and Sassa 2012a, b), and the relationship of four grain shape genes *GS3*, *GW2*, *gw5/qSW5*, and *G1F1* has been studied (Yan et al. 2011). However, the gene-to-trait pathway and relationship between genes identified by different characters are largely unknown (Huang et al. 2013).

Conventionally, the evaluation of grain shape is performed manually. In case of heavy workloads, long operating time, and short of experience, the manual measurement is less efficient and error-prone. Any mistakes in data collection may lead to incorrect and misleading results in genetic study and rice breeding. For this reason, more experienced workers and supervisors are needed to frequently check and verify the data to avoid the mistake. But subjective errors are still inevitable especially when the workers are at fatigue. Automated assessment of plant phenotypes is ideal and essential in the situation of large genetic and breeding populations. Recently, the two-dimensional (2D) digital image analysis has become available for the high-throughput phenotyping on traits like shoot biomass, yield and yield components, and grain shape (Yang et al. 2013). Taking grain shape for an example, the system can investigate more characters than GL, GW, and LW in much shorter time. However, the relationship between the conventional and novel 2D characters on grain shape is not clear. Genetic study on 2D grain shape characters is still lacking.

Our objectives in this study were (1) to investigate three conventional and four novel characters on grain shape in one *japonica* × *indica* genetic population by the 2D image analysis; (2) to study the relationship between the seven characters on grain shape; (3) to identify the genetic architecture and common stable QTL on grain shape measured by the seven characters; and (4) to discover novel QTL on grain shape which may contribute to future rice quality breeding.

## Materials and methods

### Population development and genotyping

Two parents of the genetic population used in this study are *Oryza sativa* ssp. *japonica* cv. Asominori and *Oryza sativa* ssp. *indica* cv. IR24. They were planted and the cross was firstly made in the 2007 summer season in the experimental field of Nanjing Agricultural University, Jiangsu Province, China. Their F_1_ hybrids were planted in the 2007 winter season in Sanya, Hainan Province, China, and more than 500 F_2_ seeds were harvested at maturity. The following generations were alternatively planted in the previous two locations till F_12_, when no visual segregation was observed within each line in the field. Single seed descent was applied for generation advance during the repeated selfing process. At the end, a total of 215 recombinant inbred lines (RILs) were retained, and each RIL can be traced back to an individual plant in the F_2_ generation.

DNA of each F_12_ RIL was isolated and extracted for genotyping. DNA extraction was carried out according to the procedure described by Dellaporta et al. (1983). The polymerase chain reaction (PCR) was performed using the procedure of Chen et al. (1997), with minor modifications. The protocol of PCR was briefly described as follows. The template DNA was subjected to denaturation at 94 °C for 5 min, followed by 32 cycles of PCR amplification (denaturation at 94 °C for 1 min, primer annealing at 48–55 °C for 30 s, and primer extension at 72 °C for 1 min) and a final extension at 72 °C for 5 min. The PCR products were separated through electrophoresis on an 8 % non-denaturing polyacrylamide gel and detected using the silver staining method of Sanguinetti et al. (1994). A total of 933 pairs published SSR markers (McCouch et al. 2002) were firstly screened for Asominori and IR24, and 313 markers (33.55 %) showed polymorphism between the two parents. Referring to the rice consensus map (McCouch et al. 2002), we selected 143 evenly distributed markers to screen the 215 RILs.

### Field experiments and trait measurement

The 215 RILs and their parents were grown from May to November, 2013 in four geologically and ecologically diverse locations in China, i.e., Guilin (24.18°N, 109.45°E), Guiyang (26.35°N, 106.42°E), Nanchang (28.38°N, 116.24°E), and Nanjing (31.95°N, 119.16°E). The four locations have rice as the major cultivated and consumed crop. A randomized complete block design was applied with two replications at each location. Each entry plot consisted of four rows, and each row was cultivated with ten individual plants. Field managements during the growing season were similar to those adopted by local farmers. Three representative individual plants in the central part of each plot were used to measure various grain traits. Grains of the selected plants were harvested and air-dried, and then stored at the room temperature for at least 3 months before trait measurement or investigation.

Seven grain shape characters were evaluated by SC-G rice grain appearance quality image analysis system developed by Hangzhou WSeen Detection Technology Co., Ltd, China. Firstly, all filled grains from each selected rice plant were divided into a number of samples, each having more or less 1000 grains (about ~25 g). Each sample was spread as even as possible on one 21.00 cm × 30.00 cm flat-bed surface to be photographed. The grain image was acquired by an Eloam high-speed photographic apparatus S500A3B with a resolution of 4800 × 2400 × 24 bits. The image analysis system is able to distinguish individual grains when they are randomly spread on the flat-bed surface. It can distinguish individuals even when some grains are overlapped (Zhong et al. 2009).

Grain length (GL), grain width (GW), grain circumference (GC), and grain area (GA) were firstly deduced from pixel number on the projected image. GL is defined as the maximum Euclidean distance between two boundary points of the grain, and GW is defined as the maximum length of straight lines perpendicular to the line of GL. Grain length-to-width ratio (LW) is equal to the ratio of GL and GW. Grain diameter (GD) is equal to the diameter of a cycle that has an area equal to GA, i.e., ${\text{GD}} = \sqrt {4\text{ } \times \text{ }{\text{GA}}/\pi }$. Fitting a rice grain image as an ellipse that has the same area, and uniform distribution of points bounded by the perimeter of the profile of grain image, the grain roundness is calculated by ${\text{GR}} = \frac{{4\text{ } \times \text{ }{\text{GA}}}}{{\pi \text{ } \times \text{ (}{\text{major axis}})^{2} }}$, where major axis is largest distance between antipodal points on the fitted ellipse.

### Phenotypic data analysis

ANOVA was used to test the statistical significance of various sources of variation. In the combined ANOVA across the four locations, phenotype was partitioned into overall mean, replication (i.e., block) effect per environment, genotypic effect, environment effect, genotype by environment (GE) effect, and random error effect. Let *y*_*ijk*_ be the observed value of a trait in interest for the *i*th RIL in the *k*th replication in the *j*th environment (equivalent to location in this study). The linear model used in ANOVA is therefore,

1 $$ y_{ijk} = \mu + R_{k/j} + G_{i} + E_{j} + GE_{ij} + \varepsilon_{ijk} ,\;{\text{and}}\;\varepsilon_{ijk} \,\sim \,N(0, \, \sigma_{\varepsilon }^{2} ) $$

where *i* = 1, 2, …, *n* (*n* = 215 in this study), *j* = 1, 2, …, *e* (*e* = 4 in this study), *k* = 1, 2, …, *r* (*r* = 2 in this study), $\mu$ is overall mean of the RIL population, *R*_*k/j*_ is the *k*th replication effect in the *j*th environment, *G*_*i*_ is genotypic effect of the *i*th RIL, *E*_*j*_ is environmental effect of the *j*th environment, *GE*_*ij*_ is interaction effect between the *i*th RIL and *j*th environment, and *ε*_*ijk*_ is random error effect which was assumed to be normally distributed with a mean of zero. Once the linear model of ANOVA is defined, total degree of freedom and total sum square can be partitioned into the components defined in the linear model, from which mean square (MS) of each source of variation can be calculated, and the significance test can be conducted.

Heritability is a useful genetic parameter. In the broad sense, heritability is the proportion of genetic variance compared with phenotypic variance. From the theoretical expectation of MS, genetic variance ($\sigma_{G}^{2}$), interaction variance ($\sigma_{GE}^{2}$), and error variance ($\sigma_{\varepsilon }^{2}$) can be estimated by the following equations, where *e* = 4 and *r* = 2 in this study.

2 $$ \begin{aligned}   \sigma _{G}^{2}  =  & \,\frac{1}{{e \times r}}{\text{(MS}}_{G}  - {\text{MS}}_{\varepsilon } {\text{),}} \\    \sigma _{{GE}}^{2}  =  & \,\frac{1}{r}{\text{(MS}}_{{GE}}  - {\text{MS}}_{\varepsilon } {\text{),}}\;{\text{and}}\;\sigma _{\varepsilon }^{2}  = {\text{MS}}_{\varepsilon }  \\  \end{aligned}  $$

It is generally agreed that environmental variance should not be included in the calculation of heritability (Holland et al. 2003). Phenotypic variance per plot in multi-environmental trials can be written as $\sigma_{P}^{2} = \sigma_{G}^{2} + \sigma_{GE}^{2} + \sigma_{\varepsilon }^{2}$. Therefore, the phenotypic variance on the mean performance across replications and environments can be written as $\sigma_{{\bar{P}}}^{2} = \sigma_{G}^{2} + \frac{1}{e}\sigma_{\text{GE}}^{2} + \frac{1}{er}\sigma_{\varepsilon }^{2}$. Heritability on the plot level and heritability on the mean performance of each RIL in the genetic population can be estimated from the following two equations, respectively.

3 $$ H_{P}^{2} = \frac{{\sigma_{G}^{2} }}{{\sigma_{P}^{2} }} = \frac{{\sigma_{G}^{2} }}{{\sigma_{G}^{2} + \sigma_{GE}^{2} + \sigma_{\varepsilon }^{2} }},\;{\text{and}}\;H_{{\bar{P}}}^{2} = \frac{{\sigma_{G}^{2} }}{{\sigma_{{\bar{P}}}^{2} }} = \frac{{\sigma_{G}^{2} }}{{\sigma_{G}^{2} + \tfrac{1}{e}\sigma_{GE}^{2} + \tfrac{1}{er}\sigma_{\varepsilon }^{2} }} $$

Genotypic variance is the same in calculating the two levels of heritability, but phenotypic variance is reduced in the mean performance across environments and replications. Obviously, heritability has a higher value on phenotypic mean. The method previously described has been implemented in tool “ANOVA” in the QTL IciMapping software (Meng et al. 2015).

### Genotypic data analysis

Genetic linkage map construction and QTL mapping were conducted in the QTL IciMapping software (Meng et al. 2015), which is public and freely available (http://www.isbreeding.net/software/). For map construction, physical positions of SSR markers were used in grouping as anchor information. Algorithm nnTwoOpt was used to acquire the preliminary order and positions of linked markers, where the nearest neighbor was used to construct an initial order and the two-opt algorithm in solving traveling salesman problems was used to improve the marker order. Rippling algorithm at a window size of 8 markers was used to fine-tune the linkage map with the objective to minimize the sum of adjacent recombination frequencies on each linkage map.

Inclusive Composite Interval Mapping, known as ICIM (Li et al. 2007; Wang 2009), was used for QTL identification. LOD threshold was obtained on a total of 7000 permutation tests for the seven characters and a genome-wide type I error rate at 0.05. Probabilities for entering and leaving variables were set at 0.001 and 0.002, respectively, in the stepwise regression aiming to determine the linear relationship between phenotype and marker type. This linear model was then used for background genetic variation control in ICIM QTL mapping. The scanning step was set at 1 cM across the 12 rice chromosomes. A peak in a marker interval along the LOD profile was treated as a QTL, if there is at least one environment with the peak value higher than the LOD threshold. The identified QTL was named according to McCouch et al. (1997).

## Results

### Phenotypic distribution, correlation, and ANOVA

**Fig. 1:**
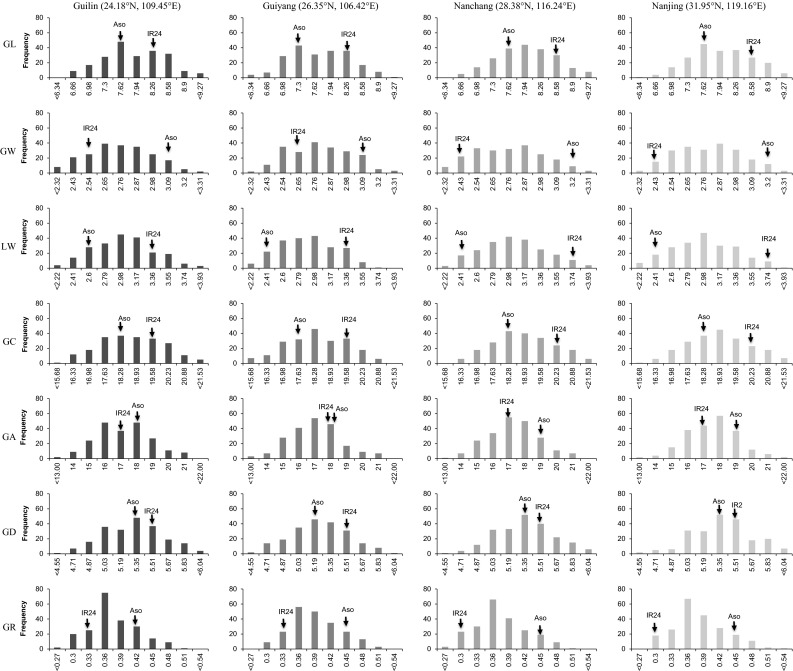
Frequency distribution of the seven characters on rice grain shape in the RIL population grown in four environments. Aso and IR24 at the top of each histogram represented the two parents *Oryza sativa* ssp. *japonica* cv. Asominori and *Oryza sativa* ssp. *indica* cv. IR24, respectively. *GL* grain length, *GW* grain width, *LW* grain length-to-width ratio, *GC* grain circumference, *GA* grain area, *GD* grain diameter, and *GR* grain roundness

Phenotypic frequency distributions of the seven characters in the RIL population and four environments are shown in Fig. 1. Difference between the two parents varies by environment. But, IR24 has consistently greater values on GL, LW, GC, and GD, and Asominori has consistently greater values on GW, GA, and GR in all environments, indicating that the investigated characters may have high stability across environments. In the RIL population, transgressive segregation at both directions can be observed, but the transgressive level is different from the seven characters (Fig. 1). Higher level of transgressive segregation can be found from GL, GA, and GD.

For the seven characters, similar correlation coefficients were observed in the four environments (Table S1). Coefficients calculated from the phenotypic mean of each RIL across the four environments and two replications are shown in Table 1. Correlation coefficients are close to or >0.9 between GL and GC, GL and GD, GC and GD, and GA and GD (Table 1). This is understandable when thinking longer rice grain is always larger in grain size. GL, GC, GD, and GA reflect size of the rice grain in different ways, and one cannot be replaced by the other one. LW and GR have a correlation coefficient close to −1, indicating they measured grain shape in two opposite ways. When the 2D image of rice grain can be approximated by an ellipse, LW is actually ratio of long axis over short axis, and GR is ratio of short axis over long axis. This explains the highly negative correlation between LW and GR.

**Table 1:** Correlation coefficient between the seven characters on rice grain shape across the four locations

Character	GL	GW	LW	GC	GA	GD	GR
GL	1.000						
GW	−0.238**	1.000					
LW	0.777**	−0.790**	1.000				
GC	0.985**	−0.078*	0.666**	1.000			
GA	0.687**	0.529**	0.083*	0.793**	1.000		
GD	0.931**	0.124**	0.501**	0.974**	0.894**	1.000	
GR	−0.771**	0.793**	−0.986**	−0.657**	−0.087*	−0.494**	1.000

*GL* grain length, *GW* grain width, *LW* grain length-to-width ratio, *GC* grain circumference, *GA* grain area, *GD* grain diameter, and *GR* grain roundness

*, ** Significance at the level of 0.05 and 0.01, respectively

For each character, ANOVA combining the four environments showed that there were significant variations from the four environments, the two replications (or two blocks) per environment, the 215 genotypes, and the genotype by environment (GE) interactions (Table S2). Significance from the two replications in the four environments indicated that the block effect should be considered in the ANOVA linear model in Eq. (1) in order to reduce the random error, which actually represents one of the three basic principles in field experimental design. Four components of variance calculated by Eq. (2) and heritability in the broad sense calculated by Eq. (3) are shown in Table 2. Obviously, environment and GE interaction had much lower variances, compared with genotype, indicating that genotypic variation was the major part in the observed phenotypic variation for the seven characters. By plot, the characters had the heritability around 0.9 (Table 2). Much less GE interactions and random errors were included in the phenotypic mean across environments and replications. Therefore, the heritability was increased when estimated by the phenotypic mean (Table 2). High heritability was also found in other studies, for example see Huang et al. 2013.

**Table 2:** Variance components and heritability for the seven characters on rice grain shape estimated in the RIL population

Character	Variance components				Heritability	
	Environment	Genotype	G by E interaction	Random error	Plot level	Genotypic mean level
GL	0.0138	0.3501	0.0139	0.0111	0.9334	0.9944
GW	0.0006	0.0452	0.0021	0.0026	0.9073	0.9918
LW	0.0038	0.1255	0.0039	0.0040	0.9403	0.9989
GC	0.0623	1.4858	0.0648	0.0542	0.9258	0.9924
GA	0.0423	2.4288	0.1637	0.1579	0.8830	0.9838
GD	0.0035	0.0824	0.0049	0.0046	0.8971	0.9885
GR	0.0001	0.0022	0.0001	0.0001	0.9448	0.9954

*GL* grain length, *GW* grain width, *LW* grain length-to-width ratio, *GC* grain circumference, *GA* grain area, *GD* grain diameter, and *GR* grain roundness

### Parental contribution, marker distortion, and linkage maps

**Fig. 2:**
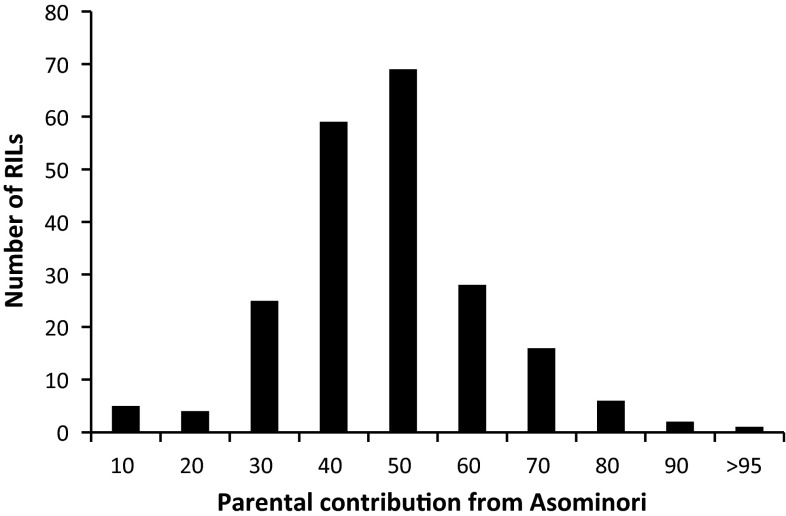
Frequency distribution of the genetic contribution from parent *Oryza sativa* spp. *japonica* cv. Asominori to each line in the RIL population

Parental contribution is the proportion of the genome contributed by a parent to its progeny (Wang and Bernardo 2000). In RIL populations, each line is homozygous, and genotypic frequency is equivalent to gene (or allele) frequency at each locus. Therefore, for each of the 215 RILs, parental contribution from Asominori can be calculated by the proportion of Asominori marker type to the total marker number. Among the 215 RILs, it can be seen from Fig. 2 that Asominori had a contribution between 35 and 65 % to 156 (or 72.56 %) lines. There were 9 (or 4.19 %) RILs where Asominori had a contribution below 25 %, and 9 (or 4.19 %) RILs where Asominori had a contribution above 85 %. As single seed descent was strictly applied during the repeated selfing in developing the RIL population, the great variation on parental contribution observed in Fig. 2 may come from the random genetic drift, which can be hardly controlled in breeding and genetic populations.

**Fig. 3:**
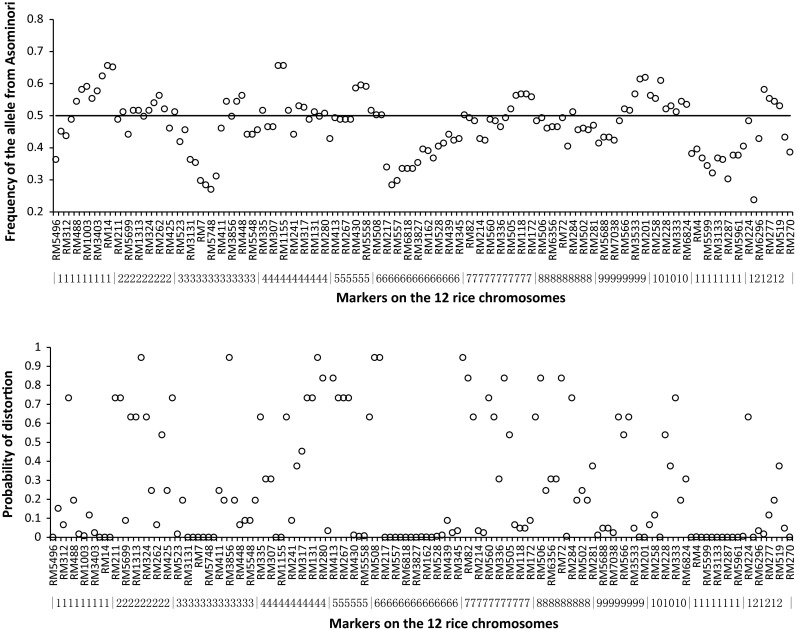
Frequency of the allele from *Oryza sativa* spp. *japonica* cv. Asominori (*upper*) and the segregation distortion test (*lower*) in the RIL population at each marker locus

Regarding the 143 SSR markers, two alleles at each locus should be fitted by the 1:1 Mendelian ratio if there is no segregation distortion in the RIL population, i.e., each allele has the expected frequency of 0.5. Observed frequency of the allele from Asominori and the segregation distortion test are shown in Fig. 3. Allele frequencies at the 143 marker loci ranged from 0.2372 to 0.6558 (upper Fig. 3), and the average was 0.4722. When the significance level of 0.001 was applied, a total of 31 markers were identified to have segregation distortion, 4 markers on chromosome 1, 6 on chromosome 3, 2 on chromosome 4, 8 on chromosome 6, 2 on chromosome 9, 9 on chromosome 11, and 2 on chromosome 12 (lower Fig. 3).

**Fig. 4:**
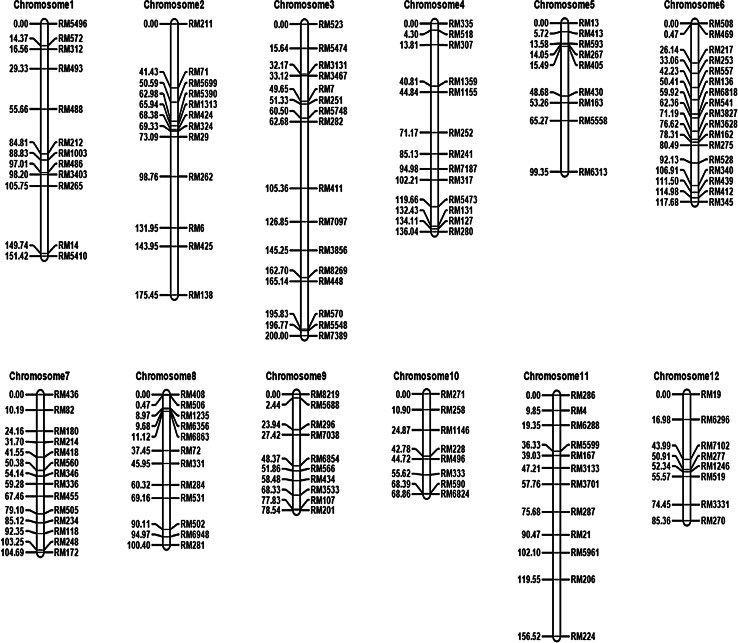
The genetic linkage map of 143 SSR markers constructed in the RIL population derived from *Oryza sativa* ssp. *japonica* cv. Asominori and *Oryza sativa* ssp. *indica* cv. IR24

Linkage maps of the 143 SSR markers constructed from the RIL population had a total length of 1474.31 cM (Fig. 4). Map length of two adjacent markers ranged from 0.24 to 32.09 cM, with an average of 10.31 cM. There were 83 marker intervals (or 58.04 %) shorter than 10 cM, and 6 intervals (or 4.20 %) longer than 25 cM. Larger gaps were observed on chromosomes 1 to 5 and 11, and markers were relatively evenly distributed on other chromosomes (Fig. 4).

### QTL identified for the seven characters

A LOD threshold of 2.65 estimated from 7000 times of permutation test was used in QTL mapping for the seven characters. Seven QTL were detected for GL on four chromosomes (Table 3). qGL2-1 and qGL3-1 were detected with positive additive effects in four environments (Table 3), indicating the allele from IR24 increased GL. qGL2-1 had the LOD score from 2.78 to 6.31, and explained 4.46-9.65 % of the variation on phenotypic mean in each environment. qGL3-1 had the highest LOD scores and explained about 50 % of the variation. Three QTL were detected in three environments, among which qGL2-2 and qGL3-2 had negative effects and qGL8-2 had positive effects (Table 3). QTL not significant in all environments also showed peaks in non-significant environments, and the additive effects were at the same direction as those in the significant environments.

**Table 3:** QTL of grain shape identified by grain length (GL) in the four environments

QTL^a^	Chr.	Left marker	Right marker	Guilin (24.18°N, 109.45°E)				Guiyang (26.35°N, 106.42°E)				Nanchang (28.38°N, 116.24°E)				Nanjing (31.95°N, 119.16°E)			
				Pos.^b^	LOD^c^	PVE (%)^d^	Add^e^	Pos.^b^	LOD^c^	PVE (%)^d^	Add^e^	Pos.^b^	LOD^c^	PVE (%)^d^	Add^e^	Pos.^b^	LOD^c^	PVE (%)^d^	Add^e^
qGL2-1	2	RM29	RM262	87	**2.78**	4.67	0.13	83	**6.31**	9.25	0.18	90	**5.20**	7.58	0.17	89	**4.40**	7.89	0.17
qGL2-2	2	RM6	RM425	140	**5.84**	7.50	−0.17	139	**5.65**	5.99	−0.15	140	**5.06**	5.95	−0.15	140	2.11	2.62	−0.10
qGL3-1	3	RM411	RM7097	112	**27.99**	49.97	0.43	112	**30.05**	48.69	0.41	110	**32.97**	54.43	0.45	111	**28.15**	50.10	0.44
qGL3-2	3	RM8269	RM448	163	**4.63**	4.86	−0.14	163	0.81	0.70	−0.05	163	**4.88**	4.47	−0.13	163	**4.47**	4.68	−0.13
qGL7	7	RM505	RM234	80	**3.16**	3.37	0.11	83	**4.54**	4.29	0.12	83	1.37	1.28	0.07	81	1.67	1.81	0.08
qGL8-1	8	RM6863	RM72	21	1.77	3.17	0.11	21	**3.51**	5.81	0.14	12	0.81	0.74	0.05	17	0.73	1.10	0.07
qGL8-2	8	RM502	RM6948	92	**5.47**	6.12	0.15	91	1.17	1.03	0.06	92	**5.97**	5.99	0.15	93	**3.29**	3.70	0.12

^a^A peak in a marker interval along the LOD profile was treated as a QTL, if there is at least one environment with the peak value higher than the LOD threshold

^b^Chromosomal position (cM) of the peak

^c^The number in bold was the value of a peak higher than the threshold value in the LOD profile

^d^Percentage of the phenotypic variation explained by the locus at the peak position on the LOD profile

^e^Additive effect of the identified QTL. Positive additive effect indicated the allele from IR24 increased grain length, and the allele from Asominori decreased grain length. Negative additive effect indicated the allele from IR24 decreased grain length, and the allele from Asominori increased grain length

Seven QTL were detected for GW (Table 4). qGW3-1 and qGW5 were detected with negative additive effects in four environments (Table 4). qGL3-1 had the LOD score from 3.03 to 4.38, explaining 5.19–7.07 % of the variation on phenotypic mean in each environment. qGL5 had the highest LOD scores and explained about 20 % of the variation.

**Table 4:** QTL of grain shape identified by grain width (GW) in the four environments

QTL^a^	Chr.	Left marker	Right marker	Guilin (24.18°N, 109.45°E)				Guiyang (26.35°N, 106.42°E)				Nanchang (28.38°N, 116.24°E)				Nanjing (31.95°N, 119.16°E)			
				Pos.^b^	LOD^c^	PVE (%)^d^	Add^e^	Pos.^b^	LOD^c^	PVE (%)^d^	Add^e^	Pos.^b^	LOD^c^	PVE (%)^d^	Add^e^	Pos.^b^	LOD^c^	PVE (%)^d^	Add^e^
qGW2-1	2	RM211	RM71	10	2.39	6.50	−0.06	23	1.47	5.38	−0.05	5	1.66	2.57	−0.04	11	**4.97**	13.56	−0.08
qGW2-2	2	RM1313	RM424	66	**3.61**	4.66	−0.05	66	0.91	1.11	−0.02	68	0.01	0.01	0.00	66	0.01	0.01	0.00
qGW2-3	2	RM29	RM262	98	1.21	1.58	−0.03	88	**3.95**	9.62	−0.07	70	**4.92**	5.58	−0.05	70	**5.45**	6.10	−0.05
qGW3-1	3	RM411	RM7097	111	**3.66**	7.07	−0.06	109	**3.03**	5.19	−0.05	109	**4.38**	6.79	−0.06	112	**4.08**	7.07	−0.06
qGW3-2	3	RM8269	RM448	165	1.74	2.13	−0.03	165	0.49	0.61	−0.02	163	1.16	1.23	−0.03	163	**3.13**	3.38	−0.04
qGW5	5	RM267	RM405	15	**19.62**	30.50	−0.12	13	**21.34**	34.97	−0.12	15	**22.59**	31.24	−0.13	15	**20.99**	27.45	−0.12
qGW6	6	RM136	RM6818	59	0.66	0.88	−0.02	59	0.49	0.64	−0.02	58	**4.44**	5.86	−0.06	59	1.55	1.73	−0.03

^a^A peak in a marker interval along the LOD profile was treated as a QTL, if there is at least one environment with the peak value higher than the LOD threshold

^b^Chromosomal position (cM) of the peak

^c^The number in bold was the value of a peak higher than the threshold value in the LOD profile

^d^Percentage of the phenotypic variation explained by the locus at the peak position on the LOD profile

^e^Additive effect of the identified QTL. Positive additive effect indicated the allele from IR24 increased grain width, and the allele from Asominori decreased grain width. Negative additive effect indicated the allele from IR24 decreased grain width, and the allele from Asominori increased grain width

Eight QTL were detected for LW (Table 5). qLW3 and qLW5 were detected with positive additive effects in four environments (Table 5). qLW3 had the highest LOD score and explained about 30 % of the variation in each environment. qLW5 had the LOD score from 15.40 to 17.33, and explained 13.88-31.71 % of the variation. qLW2-2 was detected in three environments with positive additive effects (Table 5).

**Table 5:** QTL of grain shape identified by grain length–width ratio (LW) in the four environments

QTL^a^	Chr.	Left marker	Right marker	Guilin (24.18°N, 109.45°E)				Guiyang (26.35°N, 106.42°E)				Nanchang (28.38°N, 116.24°E)				Nanjing (31.95°N, 119.16°E)			
				Pos.^b^	LOD^c^	PVE (%)^d^	Add^e^	Pos.^b^	LOD^c^	PVE (%)^d^	Add^e^	Pos.^b^	LOD^c^	PVE (%)^d^	Add^e^	Pos.^b^	LOD^c^	PVE (%)^d^	Add^e^
qLW1	1	RM488	RM212	75	1.56	2.59	0.06	74	1.13	1.68	0.04	84	1.99	1.59	0.05	84	**2.84**	2.17	0.05
qLW2-1	2	RM1313	RM424	66	**6.54**	6.21	0.09	66	0.06	0.04	0.01	66	0.08	0.06	0.01	66	**3.32**	2.41	0.06
qLW2-2	2	RM29	RM262	98	1.69	1.55	0.04	86	**8.41**	13.11	0.12	85	**9.17**	13.22	0.14	95	**4.49**	4.29	0.08
qLW3	3	RM411	RM7097	111	**19.79**	28.24	0.19	110	**26.52**	36.42	0.20	109	**24.64**	30.48	0.21	110	**25.43**	29.68	0.20
qLW4	4	RM5473	RM131	123	2.14	2.26	−0.05	121	**2.91**	2.61	−0.05	120	2.16	1.66	−0.05	122	1.77	1.38	−0.04
qLW5	5	RM267	RM405	26	**17.33**	31.71	0.20	15	**15.40**	14.99	0.13	15	**17.22**	16.13	0.15	15	**16.55**	13.88	0.14
qLW8	8	RM1235	RM6356	9	**3.49**	3.20	0.06	9	1.13	0.88	0.03	9	**2.72**	2.16	0.06	9	2.62	1.88	0.05
qLW9	9	RM434	RM3533	61	**2.68**	2.83	−0.06	66	2.38	2.14	−0.05	64	1.02	0.93	−0.04	63	1.50	1.26	−0.04

^a^A peak in a marker interval along the LOD profile was treated as a QTL, if there is at least one environment with the peak value higher than the LOD threshold

^b^Chromosomal position (cM) of the peak

^c^The number in bold was the value of a peak higher than the threshold value in the LOD profile

^d^Percentage of the phenotypic variation explained by the locus at the peak position on the LOD profile

^e^Additive effect of the identified QTL. Positive additive effect indicated the allele from IR24 increased the grain length–width ratio, and the allele from Asominori decreased the ratio. Negative additive effect indicated the allele from IR24 decreased the ratio, and the allele from Asominori increased the ratio

Six QTL were detected for GC (Table 6). qGC3-1 and qGC8 were detected with positive additive effects in four environments (Table 6). qGC3-1 had the highest LOD score and explained about 50 % of the variation in each environment. qGC8 had the LOD score from 3.87 to 6.80, and explained 4.06–7.10 % of the variation.

**Table 6:** QTL of grain shape identified by grain circumference (GC) in the four environments

QTL^a^	Chr.	Left marker	Right marker	Guilin (24.18°N, 109.45°E)				Guiyang (26.35°N, 106.42°E)				Nanchang (28.38°N, 116.24°E)				Nanjing (31.95°N, 119.16°E)			
				Pos.^b^	LOD^c^	PVE (%)^d^	Add^e^	Pos.^b^	LOD^c^	PVE (%)^d^	Add^e^	Pos.^b^	LOD^c^	PVE (%)^d^	Add^e^	Pos.^b^	LOD^c^	PVE (%)^d^	Add^e^
qGC2-1	2	RM29	RM262	89	1.48	2.46	0.20	86	**3.10**	5.06	0.28	90	**3.36**	5.36	0.29	89	2.33	4.92	0.28
qGC2-2	2	RM6	RM425	140	**7.76**	9.92	−0.40	141	**7.07**	8.32	−0.36	140	**5.65**	6.97	−0.33	140	1.55	2.16	−0.19
qGC3-1	3	RM411	RM7097	113	**27.50**	48.83	0.90	113	**28.37**	51.29	0.88	111	**31.53**	54.09	0.91	111	**26.48**	52.31	0.92
qGC3-2	3	RM8269	RM448	163	**5.86**	6.09	−0.32	163	2.05	1.94	−0.17	163	**5.87**	5.70	−0.30	163	**5.17**	6.12	−0.32
qGC7	7	RM505	RM234	81	**3.98**	4.33	0.27	82	**5.24**	5.87	0.30	84	1.88	1.77	0.17	81	2.41	2.87	0.22
qGC8	8	RM502	RM6948	92	**6.15**	6.81	0.34	91	**3.87**	4.06	0.25	92	**6.80**	7.10	0.33	93	**4.20**	5.37	0.30

^a^A peak in a marker interval along the LOD profile was treated as a QTL, if there is at least one environment with the peak value higher than the LOD threshold

^b^Chromosomal position (cM) of the peak

^c^The number in bold was the value of a peak higher than the threshold value in the LOD profile

^d^Percentage of the phenotypic variation explained by the locus at the peak position on the LOD profile

^e^Additive effect of the identified QTL. Positive additive effect indicated the allele from IR24 increased grain circumference, and the allele from Asominori decreased grain circumference. Negative additive effect indicated the allele from IR24 decreased grain circumference, and the allele from Asominori increased grain circumference

Ten QTL were detected for GA (Table 7). qGA3-1 and qGA5 were both detected in four environments, but one had positive additive effect and the other had negative effect (Table 7). qGA3-1 had the LOD score from 8.59 to 17.31, and explained 15.21–30.27 % of the variation in each environment. qGA5 had the LOD score from 5.20 to 11.29, and explained 8.58–12.44 % of the variation. Three QTL were detected in three environments, among which qGA2-2 and qGA3-2 had negative additive effects, and qGL7 had positive additive effect (Table 7).

**Table 7:** QTL of grain shape identified by grain area (GA) in the four environments QTL

QTL^a^	Chr.	Left marker	Right marker	Guilin (24.18°N, 109.45°E)				Guiyang (26.35°N, 106.42°E)				Nanchang (28.38°N, 116.24°E)				Nanjing (31.95°N, 119.16°E)			
				Pos.^b^	LOD^c^	PVE (%)^d^	Add^e^	Pos.^b^	LOD^c^	PVE (%)^d^	Add^e^	Pos.^b^	LOD^c^	PVE (%)^d^	Add^e^	Pos.^b^	LOD^c^	PVE (%)^d^	Add^e^
qGA2-1	2	RM211	RM71	19	1.72	6.93	−0.44	11	0.77	1.69	−0.21	13	0.83	2.95	−0.28	14	**3.97**	18.97	−0.70
qGA2-2	2	RM6	RM425	140	**7.61**	12.09	−0.58	139	**6.23**	7.61	−0.45	141	**6.95**	10.97	−0.53	140	1.45	2.76	−0.27
qGA3-1	3	RM411	RM7097	111	**8.59**	15.21	0.66	111	**17.31**	26.36	0.84	114	**12.33**	27.15	0.84	112	**12.14**	30.37	0.89
qGA3-2	3	RM8269	RM448	177	**8.31**	19.22	−0.74	183	2.52	5.43	−0.38	184	**6.41**	17.76	−0.68	163	**4.56**	7.39	−0.44
qGA5	5	RM267	RM405	15	**8.28**	10.89	−0.55	15	**11.69**	12.44	−0.58	15	**8.36**	11.90	−0.56	15	**5.20**	8.58	−0.47
qGA6-1	6	RM528	RM340	93	0.66	0.83	−0.16	95	**6.16**	7.50	−0.46	93	0.66	0.90	−0.16	93	0.35	0.56	−0.12
qGA6-2	6	RM439	RM412	112	1.15	1.37	0.20	112	**7.54**	7.76	0.46	112	1.12	1.45	0.20	112	0.91	1.44	0.19
qGA7	7	RM505	RM234	83	**3.77**	4.98	0.38	82	**5.32**	5.73	0.39	82	**3.54**	5.16	0.37	82	2.48	4.10	0.33
qGA8	8	RM502	RM6948	92	**6.81**	9.48	0.52	93	**6.09**	6.59	0.42	94	2.07	2.87	0.27	93	2.23	3.67	0.31
qGA10	10	RM333	RM590	68	1.65	1.96	−0.24	68	**2.90**	3.02	−0.29	68	0.91	1.17	−0.17	68	1.12	1.73	−0.21

^a^A peak in a marker interval along the LOD profile was treated as a QTL, if there is at least one environment with the peak value higher than the LOD threshold

^b^Chromosomal position (cM) of the peak

^c^The number in bold was the value of a peak higher than the threshold value in the LOD profile

^d^Percentage of the phenotypic variation explained by the locus at the peak position on the LOD profile

^e^Additive effect of the identified QTL. Positive additive effect indicated the allele from IR24 increased grain area, and the allele from Asominori decreased grain area. Negative additive effect indicated the allele from IR24 decreased grain area, and the allele from Asominori increased grain area

Six QTL were detected for GD (Table 8). Three QTL on chromosomes 2, 3, and 8 were detected in four environments, two of which had positive additive effects and one of which had negative effects in four environments (Table 8). qGD2 had the LOD score from 4.48 to 9.34, and explained 6.04–12.74 % of the variation on phenotypic mean. qGD3-1, had the highest LOD score and explained about 50 % of the variation in each environment. qGD8 had the LOD score from 2.92 to 6.98, and explained 5.50–8.33 % of the variation.

**Table 8:** QTL of grain shape identified by grain diameter (GD) in the four environments

QTL^a^	Chr.	Left marker	Right marker	Guilin (24.18°N, 109.45°E)				Guiyang (26.35°N, 106.42°E)				Nanchang (28.38°N, 116.24°E)				Nanjing (31.95°N, 119.16°E)			
				Pos.^b^	LOD^c^	PVE (%)^d^	Add^e^	Pos.^b^	LOD^c^	PVE (%)^d^	Add^e^	Pos.^b^	LOD^c^	PVE (%)^d^	Add^e^	Pos.^b^	LOD^c^	PVE (%)^d^	Add^e^
qGD2	2	RM6	RM425	141	**9.34**	12.74	−0.11	141	**7.55**	9.63	−0.09	143	**6.19**	7.52	−0.08	142	**4.48**	6.04	−0.07
qGD3-1	3	RM411	RM7097	113	**24.11**	44.52	0.21	113	**25.72**	48.27	0.21	113	**26.02**	50.85	0.21	113	**24.10**	48.11	0.21
qGD3-2	3	RM8269	RM448	163	**6.00**	6.73	−0.08	163	1.89	1.93	−0.04	164	**6.54**	7.61	−0.08	163	**5.76**	6.86	−0.08
qGD5	5	RM267	RM405	15	1.77	1.82	−0.04	15	**3.28**	3.50	−0.06	15	1.38	1.47	−0.04	15	0.35	0.39	−0.02
qGD7	7	RM505	RM234	81	**3.55**	4.19	0.06	89	**4.72**	5.94	0.07	82	2.02	2.36	0.05	81	**3.83**	4.86	0.07
qGD8	8	RM502	RM6948	92	**6.98**	8.33	0.09	92	**4.55**	5.48	0.07	93	**6.39**	8.09	0.08	91	**2.92**	3.50	0.06

^a^A peak in a marker interval along the LOD profile was treated as a QTL, if there is at least one environment with the peak value higher than the LOD threshold

^b^Chromosomal position (cM) of the peak

^c^The number in bold was the value of a peak higher than the threshold value in the LOD profile

^d^Percentage of the phenotypic variation explained by the locus at the peak position on the LOD profile

^e^Additive effect of the identified QTL. Positive additive effect indicated the allele from IR24 increased grain diameter, and the allele from Asominori decreased grain diameter. Negative additive effect indicated the allele from IR24 decreased grain diameter, and the allele from Asominori increased grain diameter

Seven QTL were detected for GR (Table 9). qGR2-1, qGR3, and qGR5 were detected with negative effects in four environments (Table 9). qGR3 had the highest LOD scores from 18.93 to 25.25, and explained 28.58–34.00 % of the variation on phenotypic mean in the four environments. qGR5 had the LOD score from 12.23 to 18.87, and explained 14.88–17.67 % of the variation. qGR2-2 was detected in three environments with negative effect, and the other three, i.e., qGR1, qGR4, and qGR8, were detected in one environment (Table 9). Due to the highly negative correlation, QTL from GR and LW had effects at the opposite directions, but they were almost identical by position, LOD score, and PVE (Tables 5 and 9).

**Table 9:** QTL of grain shape identified by grain roundness (GR) in the four environments

QTL^a^	Chr.	Left marker	Right marker	Guilin (24.18°N, 109.45°E)				Guiyang (26.35°N, 106.42°E)				Nanchang (28.38°N, 116.24°E)				Nanjing (31.95°N, 119.16°E)			
				Pos.^b^	LOD^c^	PVE (%)^d^	Add^e^	Pos.^b^	LOD^c^	PVE (%)^d^	Add^e^	Pos.^b^	LOD^c^	PVE (%)^d^	Add^e^	Pos.^b^	LOD^c^	PVE (%)^d^	Add^e^
qGR1	1	RM488	RM212	78	1.44	2.28	−0.01	74	0.81	1.20	−0.01	84	1.92	1.50	−0.01	84	**2.78**	2.29	−0.01
qGR2-1	2	RM1313	RM424	66	**5.96**	6.55	−0.01	66	**3.06**	2.53	−0.01	66	**4.33**	3.43	−0.01	66	**3.04**	2.38	−0.01
qGR2-2	2	RM29	RM262	98	1.53	1.65	−0.01	95	**3.58**	3.81	−0.01	93	**3.23**	3.83	−0.01	98	**4.60**	3.87	−0.01
qGR3	3	RM411	RM7097	109	**18.93**	28.58	−0.03	110	**25.25**	34.00	−0.03	110	**23.45**	28.86	−0.03	110	**24.64**	30.71	−0.03
qGR4	4	RM5473	RM131	121	1.18	1.32	0.01	121	**3.11**	2.81	0.01	120	2.18	1.64	0.01	121	1.34	1.10	0.01
qGR5	5	RM267	RM405	13	**12.23**	14.88	−0.02	15	**16.06**	15.83	−0.02	15	**18.87**	17.67	−0.02	15	**16.44**	15.10	−0.02
qGR8	8	RM1235	RM6356	9	2.10	2.12	−0.01	9	1.09	0.85	0.00	9	**2.84**	2.22	−0.01	9	1.92	1.42	−0.01

^a^A peak in a marker interval along the LOD profile was treated as a QTL, if there is at least one environment with the peak value higher than the LOD threshold

^b^Chromosomal position (cM) of the peak

^c^The number in bold was the value of a peak higher than the threshold value in the LOD profile

^d^Percentage of the phenotypic variation explained by the locus at the peak position on the LOD profile

^e^Additive effect of the identified QTL. Positive additive effect indicated the allele from IR24 increased grain roundness, and the allele from Asominori decreased grain roundness. Negative additive effect indicated the allele from IR24 decreased grain roundness, and the allele from Asominori increased grain roundness

### Potentially novel loci harboring QTL on grain shape

**Fig. 5:**
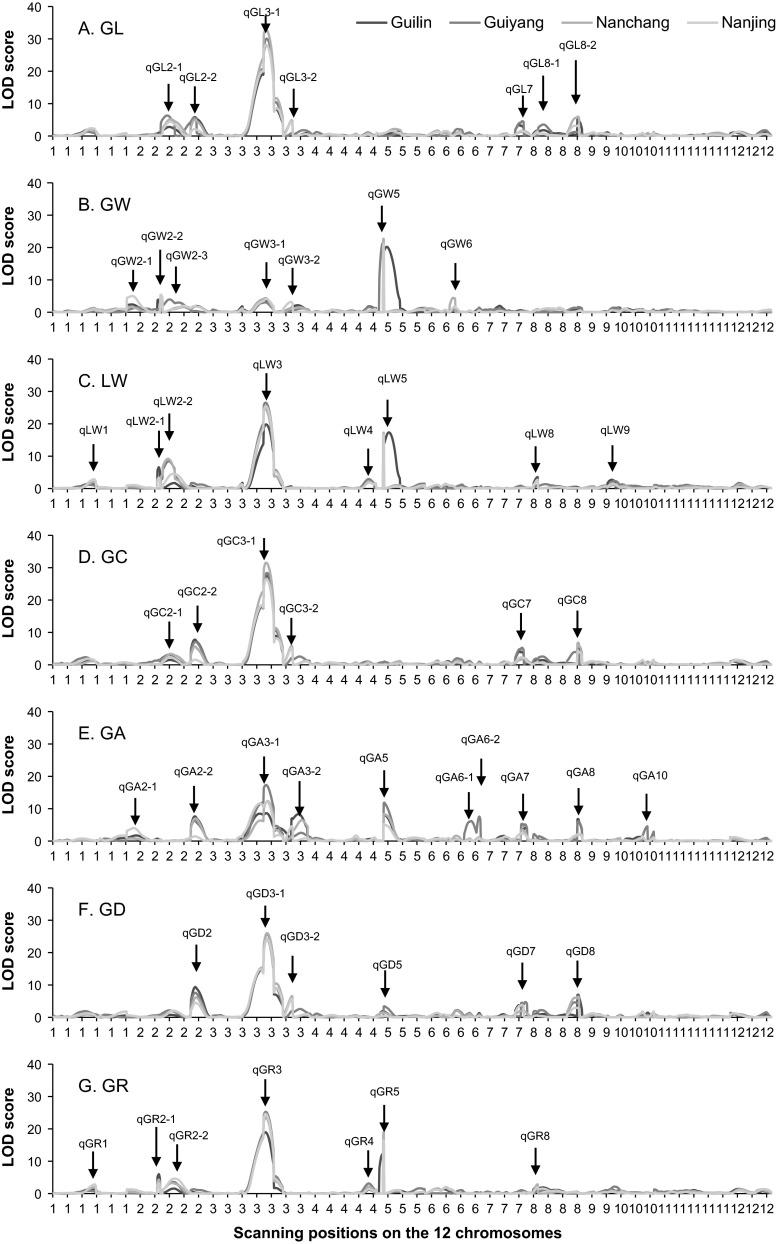
LOD profile of QTL mapping for the seven characters in four environments. Each identified QTL was indicated by an *arrow pointing* to the peak on the LOD profile. *GL* grain length, *GW* grain width, *LW* grain length-to-width ratio, *GC* grain circumference, *GA* grain area, *GD* grain diameter, and *GR* grain roundness

Combining mapping results of the seven characters (Tables 3, 4, 5, 6, 7, 8, and 9), we identified a total of 51 QTL which may affect the grain shape in various ways. Those loci were indicated by arrows on the LOD profiles shown in Fig. 5. However, due to high correlations between the seven characters (Table 1), it is highly likely that some loci have pleiotropic effects on several characters, and therefore the identified loci may not represent 51 totally different chromosomal locations. For examples, at similar position of qGL2-2, QTL was also detected for GC, GA, and GD, i.e., qGC2-2, qGA2-2, and qGD2 (Fig. 5). At similar position of qGL3-1, QTL was also detected for the other six characters, i.e., qGW3-1, qLW3, qGC3-1, qGA3-1, qGD3-,1 and qGR3 (Fig. 5). At similar position of qGW5, QTL was also detected for LW, GA, GD, and GR, i.e., qLW5, qGA5, qGD5, and qGR5 (Fig. 5). At similar position of qGL8-2, QTL was also detected for GC, GA, and GD, i.e., qGC8, qGA8, and qGD8 (Fig. 5). Each of the four chromosomal locations likely has pleiotropic effects on multiple characters, rather than closely linked loci affecting individual characters.

From Tables 3, 4, 5, 6, 7, 8, and 9 and the joint QTL mapping of four environments (results not shown), the 51 QTL were roughly clustered into 18 marker intervals on the first ten rice chromosomes (Table 10). Genes/QTL have been previously reported on 12 intervals but have not been reported on the other six (see the last column in Table 10). Detailed information on the reported genes and QTL co-located with QTL identified in this study are given in Table S3. QTL in interval RM411–RM7097 on chromosome 3 was detected by all characters in each environment, and has been fined-mapped by GL, GW, and LW, and cloned as *GS3* (Table 10). QTL in interval RM267–RM405 on chromosome 5 was detected by four characters in three environments and five characters in one environment. It has been fined-mapped by GW and LW, and cloned as *gw5/qSW5*, and *srs*-*3* (Table 10). QTL in interval RM502–RM6948 on chromosome 8 was detected by three characters in three environments and four characters in one environment. It has been fined-mapped by GW, and cloned as *qGW8/OsSPL16* (Table 10).

**Table 10:** Summary of the identified QTL in the four environments, and reported genes/QTL from previous studies

Marker interval	Chr.	Guilin (24.18°N, 109.45°E)	Guiyang (26.35°N, 106.42°E)	Nanchang (28.38°N, 116.24°E)	Nanjing (31.95°N, 119.16°E)	Reported genes/QTL
RM488–RM212	1				qLW1, qGR1	kl1.1, gl1
RM211–RM71	2				qGW2-1, qGA2-1	*GW2,* qGL-2b
RM1313–RM424	2	qLW2-1, qGW2-2, qGR2-1	qGR2-1	qGR2-1	qLW2-1, qGR2-1	Not reported
RM29–RM262	2	qGL2-1	qGL2-1, qGW2-3, qLW2-2, qGC2-1,	qGL2-1, qGW2-3, qLW2-2, qGC2-1	qGL2-1, qGW2-3, qLW2-2	qGL-2, *PGL2*, qLWR-2
RM6–RM425	2	qGL2-2, qGC2-2, qGA2-2, qGD2	qGL2-2, qGC2-2, qGA2-2, qGD2	qGL2-2, qGC2-2, qGA2-2, qGD2	qGD2	qGL-2a, *PGL2*
RM411–RM7097	3	qGL3-1, qGW3-1, qLW3, qGC3-1, qGA3-1, qGD3-1, qGR3	qGL3-1, qGW3-1, qLW3, qGC3-1, qGA3-1, qGD3-1, qGR3	qGL3-1, qGW3-1, qLW3, qGC3-1, qGA3-1, qGD3-1, qGR3	qGL3-1, qGW3-1, qLW3, qGC3-1, qGA3-1, qGD3-1, qGR3	*GS3,* qGL-3, qLWR-3, qGW-3
RM8269–RM448	3	qGL3-2, qGC3-2, qGA3-2, qGD3-2,		qGL3-2, qGC3-2, qGA3-2, qGD3-2,	qGL3-2, qGW3-2, qGC3-2, qGA3-2, qGD3-2	Not reported
RM5473–RM131	4		qLW4, qGR4			qGL-4
RM267–RM405	5	qGW5, qLW5, qGA5, qGR5	qGW5, qLW5, qGA5, qGD5, qGR5	qGW5, qLW5, qGA5, qGR5	qGW5, qLW5, qGA5, qGR5	*gw5/qSW5, srs*-*3,* qLWR-5
RM136–RM6818	6			qGW6		qGW-6
RM528–RM340	6		qGA6-1			Not reported
RM439–RM412	6		qGA6-2			Not reported
RM505–RM234	7	qGL7, qGC7, qGA7, qGD7	qGL7, qGC7, qGA7, qGD7	qGA7	qGD7	qGL7-2
RM1235–RM6356	8	qLW8		qLW8, qGR8		Not reported
RM6863–RM72	8		qGL8-1			Not reported
RM502–RM6948	8	qGL8-2, qGC8, qGA8, qGD8	qGC8, qGA8, qGD8	qGL8-2, qGC8, qGD8	qGL8-2, qGC8, qGD8	*qGW8/OsSPL16*
RM434–RM3533	9	qLW9-1				*DEP1/qPE9*-*1, SG1*
RM333–RM590	10		qGA10			kl10.1

In previous studies (Table 10), QTL on chromosome 1 has been fined-mapped by GL. The QTL in interval RM211–RM71 on chromosome 2 has been fined-mapped by GW and GL, and cloned as *GW2*. QTL in interval RM5473–RM131 on chromosome 4 has been fined-mapped by GL, QTL in interval RM136–RM6818 on chromosome 6 has been fined-mapped by GW, and QTL on chromosome 10 has been fined-mapped by GL. However, the five loci mentioned above were only detected in one environment by few characters in this study (Table 10). Considering also large size of the mapping population, high phenotyping precision of the seven characters (seen from heritability in Table 2), and high detection powers of the mapping method (Li et al. 2007, 2010; Wang 2009), we assume the six non-reported intervals may harbor novel loci on the grain shape-associated traits, and therefore are worth of further investigations. Two novel loci were located on chromosomes 2 and 3, and two each on chromosomes 6 and 8 (Table 10).

## Discussions

### Geometrical relationship of the seven characters on rice grain shape

If the 2D image of a rice grain could be fitted by an ellipse, GL is length of the long axis, GW is length of the short axis, and LW = GL/GW. In geometry, GC represents length of the circumstance which can be approximated by $\pi \,[1.5({\text{GL}} + {\text{GW}}) - \sqrt {{\text{GL}} \times {\text{GW}}} ]$, GA represents area of the ellipse which is equal to $\pi \times {\text{GL}} \times {\text{GW}}$, ${\text{GD}} = \sqrt {{\text{GL}} \times {\text{GW}}}$ (i.e., the geometrical mean of GL and GW), and GR = GW/GL (i.e., the reciprocal of LW). Therefore, it is not strange to see the correlation values shown in Table 1. For example, greater GL will result in greater LW, GC, GA, and GD, but smaller GW and GR. Therefore, GL was positively correlated with LW, GC, GA, and GD, but negatively with GW and GR (Table 1). Greater GW will result in greater GA, GD, and GR, but smaller LW and GC. Therefore, GW was positively correlated with GA, GD, and GR, but negatively with LW and GC (Table 1). LW and GR are reciprocal from each other, and a completely negative correlation was observed (Table 1).

We understand that the 2D image of a rice grain may not completely be an ellipse, and therefore GL and GW may not completely determine GC and GA, the two characters which may most suitably represent size of the rice grain, but are hardly measured manually. In this study, GL, GW, GC, and GA were directly measured in the 2-D image system. The system also output LW, GR, and GD, but LW was actually calculated from GL and GW, and GD and GR were calculated from GA.

### Advantages of the 2D image analysis in measuring grain shape

For manual measurement on grain shape, 10 or 20 filled grains were randomly selected, and then lined up lengthwise (or widthwise) along a vernier caliper in order to measure GL (or GW) (Table S3). Values of the filled grains were then averaged and used in genetic studies. The 2D image technology used in this study had advantages in measuring more characters directly, allowing a more complete description of the rice grain shape. The SC-G equipment was objective and high throughput. It takes at least 5 min to measure GL and GW of 10–20 grains manually. In comparison, SC-G can screen 800–1200 grains for the seven characters in 5–10 s. Measuring a larger number of grains from each RIL reduces the sampling errors associated with the phenotypic mean and therefore increases the estimated heritability (Table 2). In addition, the measurements from SC-G can be directly loaded into computer, so that some artificial errors in recording and transferring manual data can be greatly avoided. The image system is highly efficient in investigating grain shape characters and the WSeen product has been used in more than 300 institutes in China in recent 2 years.

In the 2D image system used in this study, GC and GA were directly measured, in addition to GL and GW. LW, GD, and GR were not directly measured, which may be called mathematically derived traits (Wang et al. 2012a, b). The use of such traits increased gene number, caused higher-order gene interactions than observed in component traits, and possibly complicated the linkage relationship between QTL as well (Wang et al. 2012a, b). The increased complexity of genetic architecture in derived traits may reduce QTL detection power and increase false discovery rate. Therefore, additional characters which can be directly measured by the 2D image system, such as GC and GA, may also contribute to more efficient and precise dissection of the genetic architecture on grain shape. If GL and GW can be viewed as one-dimensional (1D) characters, GC and GA are 2D characters. Intuitively, 2D characters may better describe the grain shape regarding the size and volume. The 2D image analysis can not only facilitate the traditional genetic studies on 1D characters, it also allows the genetic dissection of grain shape from directly measured 2D characters.

### Genetic architecture on grain shape

Grain shape is complex by phenotyping, as there is no single character which can completely quantify the shape of a grain. It is complex by genetics, as there is no single gene which can completely determine the shape of a grain. Fortunately, there are some characters which are closely associated with grain shape. These characters can be precisely measured in large scale, and have low genotype by environmental interactions, low random errors, high heritability, and high correlation relationships (Tables 1 and 2). These features explained why such a complex trait has received so much attention in rice genetics studied in past two decades.

From the seven characters investigated in the 215 RILs, a total of 51 QTL were identified to have additive effects (Tables 3, 4, 5, 6, 7, 8, and 9). Major and minor loci both exist. The major locus explained more than 20 %, while the minor locus explained a few percentage of the phenotypic variation. The identified QTL have varied stabilities across the four environments. Some were detected in four environments, but some were detected in three, two, or just one environment. Interestingly, QTL not significant in four environments also showed peaks in non-significant environments, and the additive effects were always at the same direction as those in the significant environments. This may represent another important feature of grain shape, i.e., there may not be cross-over GE interactions at the identified locus. Though each QTL has different effects in different environments, the difference will not change which allele is favorable and which allele is unfavorable. This feature of grain shape was confirmed by the low GE interactions from ANOVA (Table S2).

RIL populations are widely used in QTL mapping, where each line is homozygous in genotype and can be grown in multi-locations with replications for precision phenotyping. Additive QTL can be mapped by one-dimensional scanning, and additive by additive epistatic QTL can be mapped by 2D scanning. No dominance and dominance-associated epistasis can be studied in RIL populations. We conducted epistatic mapping (Li et al. 2008) for the 215 RIL, but did not detect significant epistatic effects. Similar results have been reported in previous studies (Huang et al. 2013). To summarize, grain shape can be precisely measured by various 1D and 2D characters, GE interaction is low, and heritability is high. It is controlled by a few major stable genes and multiple minor additive genes.

### Novelty of the six non-reported chromosomal intervals

The 51 QTL on the seven characters were clustered into 18 chromosomal intervals flanked by SSR markers (Table 10). We went through previous literatures for QTL and genes on grain shape, and compared with QTL identified in this study by physical locations or associated markers (Tables 10 and S3). Genes/QTL have been reported in 12 intervals but not yet in the other six intervals. Obviously, intervals showing QTL from multiple characters and multiple environments have been previously reported (Table 10). But this is not always the case, for example see intervals RM488–RM212 on chromosome 1, RM211–RM71 on chromosome 2, RM5473–RM131 on chromosome 4, RM136–RM6818 on chromosome 6, and RM333–RM590 on chromosome 10. We assume the six non-reported intervals may harbor novel loci on grain shape, which are worth of further investigations. Three most promising intervals are RM1313–RM424 on chromosome 2, RM8269–RM448 on chromosome 3, and RM1235–RM6356 on chromosome 8, where QTL showed up for multiple characters and in multiple environments.

Of course, we cannot exclude the possibility that some of the 51 QTL may be false positives. This problem cannot be solved in the current mapping population. In the meantime of developing the RIL population, we also developed two-way chromosome segment substation lines (CSSLs). We are using CSSLs to confirm QTL identified in the RIL population.

#### Author contribution statement

C. Yin developed the genetic population, conducted the SSR genotyping and field experiments, constructed the genetic linkage map, conducted the QTL analysis, and wrote the manuscript draft; H. Li and S. Li conducted phenotypic and genotypic data analysis; L. Xu and Z. Zhao investigated the 2D characters of rice grain in the genetic population; J. Wang designed the research, and finalized the manuscript.

## Electronic supplementary material

Supplementary material 1 (DOCX 44 kb)
