# Supplementary material for: Genetic dissection on rice grain shape by the two-dimensional image analysis in one japonica × indica population consisting of recombinant inbred lines
Source: Theor Appl Genet. 2015 Jul 2;128(10):1969–86. doi: 10.1007/s00122-015-2560-7 (PMC4572083; doi:10.1007/s00122-015-2560-7)
Supplement: Supplementary file 1 — Supplementary material 1 (DOCX 44 kb) [file 122_2015_2560_MOESM1_ESM.docx]

**Supplementary Materials**

Table S1 Correlation coefficient between the seven characters on rice grain shape in each of the four environments

| Environment | Character^a^ | GL | GW | LW | GC | GA | GD | GR |
| --- | --- | --- | --- | --- | --- | --- | --- | --- |
| Guilin (24.18°N, 109.45°E) | GL | 1.000 |  |  |  |  |  |  |
|  | GW | -0.178** | 1.000 |  |  |  |  |  |
|  | LW | 0.758** | -0.769** | 1.000 |  |  |  |  |
|  | GC | 0.985** | -0.020 | 0.647** | 1.000 |  |  |  |
|  | GA | 0.698** | 0.568** | 0.069 | 0.800** | 1.000 |  |  |
|  | GD | 0.934** | 0.174* | 0.485** | 0.975** | 0.897** | 1.000 |  |
|  | GR | -0.754** | 0.771** | -0.986** | -0.641** | -0.076 | -0.481** | 1.000 |
| Guiyang (26.35°N, 106.42°E) | GL | 1.000 |  |  |  |  |  |  |
|  | GW | -0.198** | 1.000 |  |  |  |  |  |
|  | LW | 0.776** | -0.767** | 1.000 |  |  |  |  |
|  | GC | 0.985** | -0.037 | 0.665** | 1.000 |  |  |  |
|  | GA | 0.726** | 0.520** | 0.135* | 0.827** | 1.000 |  |  |
|  | GD | 0.936** | 0.154* | 0.509** | 0.979** | 0.916** | 1.000 |  |
|  | GR | -0.766** | 0.773** | -0.988** | -0.653** | -0.132 | -0.497** | 1.000 |
| Nanchang (28.38°N, 116.24°E) | GL | 1.000 |  |  |  |  |  |  |
|  | GW | -0.274** | 1.000 |  |  |  |  |  |
|  | LW | 0.769** | -0.819** | 1.000 |  |  |  |  |
|  | GC | 0.984** | -0.107 | 0.651** | 1.000 |  |  |  |
|  | GA | 0.641** | 0.550** | 0.010 | 0.758** | 1.000 |  |  |
|  | GD | 0.923** | 0.115 | 0.468** | 0.973** | 0.879** | 1.000 |  |
|  | GR | -0.765** | 0.820** | -0.986** | -0.645** | -0.017 | -0.463** | 1.000 |
| Nanjing (31.95°N, 119.16°E) | GL | 1.000 |  |  |  |  |  |  |
|  | GW | -0.288** | 1.000 |  |  |  |  |  |
|  | LW | 0.795** | -0.803** | 1.000 |  |  |  |  |
|  | GC | 0.983** | -0.125 | 0.684** | 1.000 |  |  |  |
|  | GA | 0.679** | 0.494** | 0.101 | 0.788** | 1.000 |  |  |
|  | GD | 0.925** | 0.075 | 0.520** | 0.969** | 0.887** | 1.000 |  |
|  | GR | -0.789** | 0.805** | -0.985** | -0.675** | -0.107 | -0.514** | 1.000 |

*, **Significance at the level of 0.05 and 0.01, respectively. ^a^GL, grain length; GW, grain width; LW, grain length-to-width ratio; GC, grain circumference; GA, grain area; GD, grain diameter; and GR, grain roundness.

Table S2 Analysis of variance across the four environments in the RIL population.

| Character^a^ | Source | DF^b^ | SS | MS | F-value | Significance |
| --- | --- | --- | --- | --- | --- | --- |
| GL | Environment | 3 | 17.5260 | 5.8420 | 524.2661 | *** |
|  | Replication/Env. | 4 | 0.2715 | 0.0679 | 6.0914 | *** |
|  | Genotype | 214 | 591.9323 | 2.7660 | 248.2269 | *** |
|  | Geno×Env | 634 | 30.4682 | 0.0481 | 4.3127 | *** |
|  | Error | 836 | 9.3157 | 0.0111 |  |  |
| GW | Environment | 3 | 0.7325 | 0.2442 | 95.5493 | *** |
|  | Replication/Env. | 4 | 0.0369 | 0.0092 | 3.6117 | ** |
|  | Genotype | 214 | 76.5868 | 0.3579 | 140.0447 | *** |
|  | Geno×Env | 634 | 5.0980 | 0.0080 | 3.1466 | *** |
|  | Error | 836 | 2.1364 | 0.0026 |  |  |
| LW | Environment | 3 | 4.8852 | 1.6284 | 405.2882 | *** |
|  | Replication/Env. | 4 | 0.0908 | 0.0227 | 5.6479 | *** |
|  | Genotype | 214 | 212.1737 | 0.9915 | 246.7615 | *** |
|  | Geno×Env | 634 | 9.2121 | 0.0145 | 3.6163 | *** |
|  | Error | 836 | 3.3590 | 0.0040 |  |  |
| GC | Environment | 3 | 79.2416 | 26.4140 | 487.0133 | *** |
|  | Replication/Env. | 4 | 1.0154 | 0.2539 | 4.6805 | *** |
|  | Genotype | 214 | 2513.877 | 11.747 | 216.5904 | *** |
|  | Geno×Env | 634 | 143.8176 | 0.2268 | 4.1825 | *** |
|  | Error | 836 | 45.3416 | 0.0542 |  |  |
| GA | Environment | 3 | 54.1963 | 18.065 | 114.3969 | *** |
|  | Replication/Env. | 4 | 2.2295 | 0.5574 | 3.5295 | ** |
|  | Genotype | 214 | 4124.1410 | 19.272 | 122.0352 | *** |
|  | Geno×Env | 634 | 376.7434 | 0.5942 | 3.7629 | *** |
|  | Error | 836 | 132.0203 | 0.1579 |  |  |
| GD | Environment | 3 | 4.4818 | 1.4939 | 325.7703 | *** |
|  | Replication/Env. | 4 | 0.0611 | 0.0153 | 3.3284 | * |
|  | Genotype | 214 | 139.7135 | 0.6529 | 142.3651 | *** |
|  | Geno×Env | 634 | 11.1256 | 0.0175 | 3.8266 | *** |
|  | Error | 836 | 3.8338 | 0.0046 |  |  |
| GR | Environment | 3 | 0.0700 | 0.0233 | 312.7206 | *** |
|  | Replication/Env. | 4 | 0.0019 | 0.0005 | 6.3742 | *** |
|  | Genotype | 214 | 3.7198 | 0.0174 | 232.9188 | *** |
|  | Geno×Env | 634 | 0.1383 | 0.0002 | 2.9221 | *** |
|  | Error | 836 | 0.0624 | 0.0001 |  |  |

*, **, ***Significant at the level of 0.05, 0.01 and 0.001, respectively. ^a^GL, grain length; GW, grain width; LW, grain length-to-width ratio; GC, grain circumference; GA, grain area; GD, grain diameter; and GR, grain roundness. ^b^In the case of no missing data, DF_Env_=*e*-1=3, DF_Rep_=(*r*-1)×*e*=4, DF_Geno_=(*n*-1)=214, DF_GbyE_=(*n*-1)×(*e*-1)=642, DF_Total_=*n*×*e*×*r*-1=1719, and DF_Error_ = DF_Total_ -DF_Env_ - DF_Geno_ - DF_GbyE_ =856. In our data, there are 28 missing values, resulting in 8 GE interactions non-estimated. Therefore DF_GbyE_=642-8=634, DF_Total_= 1719-28=1691, and DF_Error_ = DF_Total_ -DF_Env_ - DF_Geno_ - DF_GbyE_ =836.

Table S3 Detailed information on reported genes and QTL which are co-located with QTL identified in this study. Manual measurement was used, unless specified. The formation not available in the literatures was indicated by "-".

| Gene/QTL | Associated markers | Physical position^a^ | Parents of mapping population^b^ | Type of mapping population | Population size | LOD threshold | Sampling and measurement | Reference |
| --- | --- | --- | --- | --- | --- | --- | --- | --- |
| gl1 | R2201, RM212 | 33053493-33053654 (Nip.) | Zhenshan97 (I), Ming63 (I) | RIL | 241 | 2.40 | 20 randomly chosen grains each plot | Xing et al. (2001) |
| kl1.1 | RM212, RZ801, RZ513 | 33053493-33053654 (Nip.) | V20A(I)/IRGC103544 (*O. glaberrima S.*) | BC3F1 | 308 | 2.89 | 10 grains each plant | Li et al. (2004) |
| kl10.1 | CDO250, BCD386, RZ421 | 19022958-22493127 (Nip.) | V20A (I)/IRGC103544 (*O. glaberrima S.*) | BC3F1 | 308 | 2.89 | 10 grains each plant | Li et al. (2004) |
| *PGL2* | - | 31423973-31424983 (Nip.) | Nipponbare (J) | - | - | - | - | Heang and Sassa (2012) |
| qGL-2 | C601, R3393 | 28351861-30273365 (Nip.) | Asonimori (J), IR24(I) | RIL, BC3F1 | 71, 66 | 3.00 | 20 fully filled paddy grains each lines, manual | Wan et al. (2005) |
| qGL-2a | C560, C1408 | 30068383-32036692 (Nip.) | Nipponbare (J), Kasalath (I) | BIL | 98 | 2.00 | 10 randomly unbroken milled grians each line | Li et al. (2003) |
| qGL-2b | G1327, C421 | 4141510-4525383 (Nip.) | Nipponbare (J), Kasalath (I) | BIL | 98 | 2.00 | 10 randomly unbroken milled grians each line | Li et al. (2003) |
| qGL-3 | R19, C1677 | 15644274-24595466 (Nip.) | Asonimori (J), IR24 (I) | RIL, BC3F1 | 71, 66 | 3.00 | 20 fully filled paddy grains each lines | Wan et al. (2005) |
| qGL-4 | XNpb331,C335 | 28905788-29156010 (Nip.) | Asonimori (J), IR24 (I) | RIL, BC3F1 | 71, 66 | 3.00 | 20 fully filled paddy grains each lines | Wan et al. (2005) |
| qGL7 | RID711, RM6389 | 28277213-28277417 (Nip.) | Nanyangzhan (J), Chuan7 (I) | RIL | 185 | 2.80 | 20 fully filled paddy grains each lines | Bai et al. (2010) |
| qGL7-2 | RM351, RM234, RM21945 | - | D50 (J), HB277 (I) | RIL | 190 | 3.00 | 20 randomly paddy grains each lines | Shao et al. (2010) |
| *GS3* | GS09, MRG5881 | 17361501-17367109 (Nip.) | Minghui63(I), Chuan7 I) | BC3F2 | 201 | 3.00 | 10 randomly chosen grains each plant | Fan et al. (2001) |
| *DEP1/qPE9-1* | RM3770, RM7424, H90, RM5652 | 16410553-16414701 (Nip.) | Wuyujing8 (J), Nongken57(J) | DH | 154 | - | 10 randomly grains each plant | Yan et al. (2007); Huang et al. (2009) |
| *SG1* | Os09g0459200 | 17350940-17352413 (Nip.) | Nippombare(J), T65(J) | activation-tagging lines | 13000 | - | 30 measurements done with a digital camera system α-100 (sony) | Nakagawa et al. (2012) |
| *srs-3* | 5-3000, 5-3247 | 3207517-3210183 (Nip.) | TCM1173 (J), Kasalath (I) | F2 | 1000 | - | - | Tanabe et al. (2007); Kitagawa et al. (2010) |
| qLWR-2 | XNpb67,XNpb132 | 18495134-22596902 (Nip.) | Asonimori (J), IR24(I) | RIL, BC3F1 | 71, 66 | 3.00 | 20 fully filled paddy grains each lines | Wan et al. (2005) |
| qLWR-3 | R19, C1677 | 15644274-24595466 (Nip.) | Asonimori (J), IR24 (I) | RIL, BC3F1 | 71, 66 | 3.00 | 20 fully filled paddy grains each lines | Wan et al. (2005) |
| qLWR-5 | R3166， R569 | 1861366-6700408 (Nip.) | Asonimori (J), IR24(I) | RIL, BC3F1 | 71, 66 | 3.00 | 20 fully filled paddy grains each lines | Wan et al. (2005) |
| qLWR-5b | R1436, R2289 | 18252951-18993336 (Nip.) | Nipponbare (J), Kasalath (I) | BIL | 98 | 2.00 | 10 randomly unbroken milled grians each line | Li et al. (2003) |
| *GW2* | RM5897, RM2634, W236, W239 | 5727458-5727615 (9311) | WY3 (J), Fengaizhan-1 (I) | BC2F2 | 190 | - | - | Song et al. (2007) |
| *gw5/qSW5* | RM3328, RMw513 | 5727083-5727615 (9311) | Nipponbare (J), Kasalath(I) Asonimori (J), IR24 (I) | F2, CSSL | 186, 66 | 3.00 | 20 fully filled paddy grains each lines | Wan et al. (2008); Weng et al. (2008); Shomura et al. (2008) |
| qGW-3 | C332, C80 | 17713671-33619269 (Nip.) | Nipponbare (J), Kasalath (I) | BIL | 98 | 2.00 | 10 randomly unbroken milled grians each line | Li et al. (2003) |
| qGW-5b | C249, R566 | 5915709-7810160 (Nip.) | Nipponbare (J), Kasalath (I) | BIL | 98 | 2.00 | 10 randomly unbroken milled grians each line | Li et al. (2003) |
| qGW-6 | C991, XNpb12 | 13684438-26162401 (Nip.) | Asonimori (J), IR24(I) | RIL, BC3F1 | 71, 66 | 3.00 | 20 fully filled paddy grains each lines | Wan et al. (2005) |
| *qGW8/OsSPL16* | RM502, PSM711 | 26501167-26506198 (Nip.) | Basmati385 (I, donor), HJX74(I) | CSSL | 153 | - | - | Wang et al. (2012) |

^a^Nip. in the bracket means the physical position is from the nipponbare sequence; 9311 in the bracket means the physical position is from the 9311 sequence. ^b^*Indica* and *japonica* parents were indicated by I and J in brackets, respectively.
